# Cost-effectiveness of Artificial Intelligence as a Decision-Support System Applied to the Detection and Grading of Melanoma, Dental Caries, and Diabetic Retinopathy

**DOI:** 10.1001/jamanetworkopen.2022.0269

**Published:** 2022-03-15

**Authors:** Jesus Gomez Rossi, Natalia Rojas-Perilla, Joachim Krois, Falk Schwendicke

**Affiliations:** 1 Department of Oral Diagnostics, Digital Health and Health Services Research, Charité–Universitätsmedizin Berlin, Berlin, Germany; 2 Department of Economics, Freie Universität Berlin, Germany; 3 Department of Analytics in the Digital Era, United Arab Emirates University, Al Ain, United Arab Emirates

## Abstract

**Objective:**

To assess the cost-effectiveness of artificial intelligence (AI) for supporting clinicians in detecting and grading diseases in dermatology, dentistry, and ophthalmology.

**Importance:**

AI has been referred to as a facilitator for more precise, personalized, and safer health care, and AI algorithms have been reported to have diagnostic accuracies at or above the average physician in dermatology, dentistry, and ophthalmology.

**Design, Setting, and Participants:**

This economic evaluation analyzed data from 3 Markov models used in previous cost-effectiveness studies that were adapted to compare AI vs standard of care to detect melanoma on skin photographs, dental caries on radiographs, and diabetic retinopathy on retina fundus imaging. The general US and German population aged 50 and 12 years, respectively, as well as individuals with diabetes in Brazil aged 40 years were modeled over their lifetime. Monte Carlo microsimulations and sensitivity analyses were used to capture lifetime efficacy and costs. An annual cycle length was chosen. Data were analyzed between February 2021 and August 2021.

**Exposure:**

AI vs standard of care.

**Main Outcomes and Measures:**

Association of AI with tooth retention–years for dentistry and quality-adjusted life-years (QALYs) for individuals in dermatology and ophthalmology; diagnostic costs.

**Results:**

In 1000 microsimulations with 1000 random samples, AI as a diagnostic-support system showed limited cost-savings and gains in tooth retention–years and QALYs. In dermatology, AI showed mean costs of $750 (95% CI, $608-$970) and was associated with 86.5 QALYs (95% CI, 84.9-87.9 QALYs), while the control showed higher costs $759 (95% CI, $618-$970) with similar QALY outcome. In dentistry, AI accumulated costs of €320 (95% CI, €299-€341) (purchasing power parity [PPP] conversion, $429 [95% CI, $400-$458]) with 62.4 years per tooth retention (95% CI, 60.7-65.1 years). The control was associated with higher cost, €342 (95% CI, €318-€368) (PPP, $458; 95% CI, $426-$493) and fewer tooth retention–years (60.9 years; 95% CI, 60.5-63.1 years). In ophthalmology, AI accrued costs of R $1321 (95% CI, R $1283-R $1364) (PPP, $559; 95% CI, $543-$577) at 8.4 QALYs (95% CI, 8.0-8.7 QALYs), while the control was less expensive (R $1260; 95% CI, R $1222-R $1303) (PPP, $533; 95% CI, $517-$551) and associated with similar QALYs. Dominance in favor of AI was dependent on small differences in the fee paid for the service and the treatment assumed after diagnosis. The fee paid for AI was a factor in patient preferences in cost-effectiveness between strategies.

**Conclusions and Relevance:**

The findings of this study suggest that marginal improvements in diagnostic accuracy when using AI may translate into a marginal improvement in outcomes. The current evidence supporting AI as decision support from a cost-effectiveness perspective is limited; AI should be evaluated on a case-specific basis to capture not only differences in costs and payment mechanisms but also treatment after diagnosis.

## Key Points

**Question:**

Are existing artificial intelligence (AI) algorithms cost-effective for use as a decision-support system in dermatology, dentistry, and ophthalmology?

**Findings:**

In this economic evaluation analyzing data from 3 Markov models used in previous cost-effectiveness studies, the use of AI was associated with a modest improvement in outcomes. All benefits were highly dependent on treatment effects assumed after diagnosis and were very sensitive to the fee paid for the use of AI.

**Meaning:**

These results suggest that even when AI can achieve better diagnostic capacities than the average physician, this may not directly translate to better or cheaper care, and that analysis using this technology should be used on a case-by-case basis.

## Teaser

This economic evaluation examines the cost-effectiveness of artificial intelligence (AI) assistance in common diagnostic tests in dermatology, dentistry, and ophthalmology compared with specialist diagnosis without AI assistance.

## Introduction

Artificial intelligence (AI) is frequently referred to as a facilitator for more precise, personalized, and safer health care.^1,2^ A major use of AI is decision support (ie, to help physicians detecting and grading diseases, such as through image analysis of skin photographs).^3^ AI algorithms with diagnostic accuracies at or above the average physician have been reported in dermatology,^4^ dentistry,^5^ and ophthalmology,^6^ among others.

Although US regulatory bodies (including the Food and Drug Administration [FDA]) approved the first AI diagnostic solution for the detection of diabetic retinopathy in 2018,^7^ the benefits that this technology could generate on existing treatment paths have not been thoroughly assessed.^8,9^ AI diagnostic solutions are currently under study in real-world settings in the US, India, Thailand, China, Australia,^10^ and Singapore.^11^ Importantly, these studies frequently take a third-party perspective and do not extrapolate over patient lifetime. Furthermore, differences between the setting in which an AI solution is deployed and where it is developed could open new questions of cost-effectiveness relevant to discussions of ever-rising health care costs.^12^ New research is necessary to determine if AI can reduce costs and improve outcomes on its own, or if it may even increase pressure on existing resources.^13^ An informed understanding can help decide possible reimbursement for the use of AI in diagnosis and to steer research and development to where most health and economic benefits can be expected.^14^

It is likely that the cost-effectiveness of AI depends on its diagnostic accuracy for the use case assumed (ie, Is it helping doctors or patients? What is the current standard of screening for the disease?), the patient population (What is the prevalence and costs of treatment for the disease studied?), and factors specific to the health care setting (What is the frequency of testing? What treatments do patients receive at each stage of the disease after being diagnosed?).

To the best of our knowledge, no previous study has modeled cost-effectiveness of existing AI algorithms for different use cases in different settings.^15^ We aimed to evaluate AI’s cost-effectiveness as a diagnostic support system in dermatology, dentistry, and ophthalmology in different countries using health economic modeling via Markov models with a lifetime horizon. We decided to account for AI as fee-for-service and explored how it factored into cost-effectiveness (per-person) through sensitivity analysis. Our research goal was to test the assumption that an AI with superior diagnostic accuracy used as a decision-support system would always clearly reduce costs and improve outcomes. Better understanding these aspects is particularly important for decision-makers assessing AI solutions, as well as for developers deciding to invest resources in decision-support systems using AI.

## Methods

### Study Design

Three model-based cost-effectiveness analyses were performed from the payer perspective for 3 diagnostic procedures in different medical disciplines—melanoma detection in dermatology, caries detection in dentistry, and detection of diabetic retinopathy in ophthalmology. AI as a diagnostic support system has been used previously to help detect and/or grade melanoma lesions on skin photography^4^; dental caries lesions on radiographs^16^; and diabetic retinopathy on fundus photography.^17^ Our economic evaluations used data and models of previously published studies that had performed cost-effectiveness analyses on each use case without involving AI (Table). In all cases, the sensitivity and specificity of AI as a diagnostic support system were compared with those of the standard of care.

**Table.** Comparative Summary of Included Models

	Dermatology	Dentistry	Ophthalmology
**Model characteristics**			
Economic model source	Losina et al^18^	Schwendicke et al^19^	Ben et al^20^
AI accuracy model	Brinker et al^4^	Cantu et al^21^	Abramoff et al^22^
Target population	General population, age 50 y	Children, age 12 y	Individuals with diabetes, age >40 y
Perspective of payer	OOP	Third-party plus OOP	Third-party
AI use-case assumption	Decision support	Decision support	Decision support
Comparator	Standard dermatological screening	Standard dental screening	Standard ophthalmological screening
Setting and location	US	Germany	Brazil
Model utilized	Markov	Markov	Markov
AI development team location	Germany	Germany	US
Fee-for-use of AI^a^	US $8	€8	R $8
Measurement of outcomes	QALY/survival	Tooth-retention	QALY
Discounting^a^	3%	3%	3%
Study perspective	Lifetime	Lifetime	Lifetime
Currency and conversion	US$	Euro (€)	R$ transformed via PPP to US$
Opportunity costs	Not considered	Not considered	Not considered
**Results (1000 microsimulations with 1000 random samples)**			
AI			
Mean cost (95% CI)	US $750.35 ($608.77-$970.95)	€320.40 (€299-€341)	R $1321 (R $1283-R $1364)
2020 PPP (95% CI)	NA	$429.49 ($400.80-$458.76)	$559 ($543-$577)
QALYs (95% CI)	86.6 (84.9-88.0)	62.4 (61.6-63.1)^c^	8.42 (8.33-8.51)
Standard			
Mean cost (95% CI)	US $759.03 ($617.64-$980.73)	€342.24 (€318-€368)	R $1260.28 (R $1222-R $1303)
2020 PPP (95% CI)	NA	$458 ($426-$493)	$533 ($517-$551)
QALYs (95% CI)	86.6 (84.9-88.0)	60.9 (60.0-61.8)^c^	8.42 (8.33-8.51)

Abbreviations: AI, artificial intelligence; NA, not applicable; OOP, out-of-pocket; PPP, purchasing-power-parity; QALY, quality-adjusted life years; R$, Brazilian real.

^a^ Explored in sensitivity analysis.

^b^ 95% CIs ranged from 2.5% to 97.5% percentiles.

^c^ Measured in tooth retention years as equivalent of QALYs.

The 3 use cases, AI applications, and health economic models are summarized in the Table in line with the Consolidated Health Economic Evaluation Reporting Standards (CHEERS) reporting guideline. Transitions between states and transition probabilities are explained in detail in eAppendices 1 to 3 in the Supplement. The settings of the different studies were the US for melanoma, Germany for caries detection, and Brazil for ophthalmology, with all parameters such as prevalence and life expectancy adjusted to these settings. Only 1 study considered the research and development costs of the AI application, which we extrapolated to the other 2 use cases, as is common practice in pharmacoeconomics.^23^ We explored in a sensitivity analysis the effects of price variation. All economic models were constructed using Markov chains with simulations at discrete yearly intervals under a lifetime horizon. No approval by an ethics committee was requested as we performed a modeling exercise; data were deidentified and no original data were used.

### Setting and Population

All 3 analyses adopted a payer perspective in 3 different health care settings. The US health care system is ranked as first in health care expenditure worldwide.^24^ Expenditures are financed by a combination of voluntary health insurance, employer insurance, and out-of-pocket expenditures, with exceptions controlled by the government for older, disabled, and low-income populations.^25^ In Germany, medical insurance, including dentistry, is 2-tiered, with most individuals (ie, over 87%) being publicly insured and only a minority being privately insured.^26^ For members using public insurance, nearly all procedures are fully covered, while only some treatments are partially or fully paid out-of-pocket.^27^ Brazil’s universal public health care system is tax-funded by federal, state, and municipal governments and, despite limitations, offers comprehensive health coverage to the majority of its population.^28^

For the dermatological use case, direct costs to the health care payer in the US system (ie, health care system costs and patients’ copayments combined) that would arise in the detection step, possible histological validation, and possible treatments and follow-up treatments were considered. Two cohorts of patients (AI vs control) entered the model to calculate morbidity, mortality, and costs. Individuals in both cohorts were in full health initially. The model assessed their risk of developing, being diagnosed, and being treated for melanoma by dermatologists, with the only difference between groups being the assistance of AI support.

For the dental use case, costs arising in the statutory German insurance as well as copayments by private insurance or out-of-pocket costs were considered, including detection costs with and without AI support and lifetime treatment and re-treatment costs. The unit of analysis was the tooth; both teeth that were sound or with an initial or advanced caries lesion were included, according to prevalence data drawn from a previous study.^29^

For the ophthalmological use case, a Brazilian taxpayer’s perspective was taken. All costs accrued by the economic model, including treatment, were covered by the Brazilian National Health Service. We included in our model a group of patients with type II diabetes at risk of developing some form of diabetic retinopathy. Participants were tested biannually.

### Comparators

For dermatology, the control group (ie, without AI) received the standard evaluation by dermatologists using a dermatoscope; accuracy for this group was extracted from previous studies.^30^ Included treatments were derived from the health economic model that was used as a reference in our study.^18^ The test group (AI) consisted of a convolutional neural network (CNN) for classifying skin photographs trained on 12 378 dermoscopic images labeled by 145 dermatologists.^4^

For dentistry, the control group was the detection of proximal caries lesion using biannual visual-tactile assessment and bitewing radiographs taken twice annually by dentists,^19^ following to the health economic model that was used as reference.^31^ In the test group, radiographic caries detection was assumed to be AI-assisted using a CNN that had been trained on 3293 images, validated on 252 images, and tested on 141 images (each of which had been labeled by 4 experts).^11^

For ophthalmology, the control group was the standard screening of diabetic retinopathy undertaken by ophthalmologists in Brazil,^20^ in line with the economic model used as a reference for the study.^32^ Diagnostic accuracy was modeled on the analysis of digital fundus photography previously used in the economic evaluation used as our data source.^33^ The test group was a CNN trained on over 1 million lesions labeled according to a framework for automated lesion detection in retinal images.^34^

### Models and Assumptions

For all 3 Markov models, initial and follow-up health states were included, with costs and utilities accrued for each transition. In the dermatological model, patients entered the model at age 48 years. In the case of dentistry, patients entered the model at age 12 years under the assumption that their permanent dentition is fully developed by then. In the case of ophthalmology, a population of individuals with diabetes entered the model at age 40 years, because according to US Centers for Disease Control and Prevention guidelines expanded screening strategies appear to be justified at that age.^35^

All models took a lifetime horizon according to their setting. In the case of melanoma, we differentiated between the risk of death to melanoma and the overall risk of death. In the case of dentistry, we followed tooth retention over average life expectancy, as tooth loss is an event that can be almost completely averted throughout a lifetime. In the case of ophthalmology, we reflected the utility derived from each stage, as diabetic retinopathy is a nonlethal disease that has a high impact on quality of life. In all cases, the development of a disease and its progression were modeled according to probabilities extracted from meta-analyses reflected in previously published peer-reviewed models. After diagnosis and treatment, the models transitioned patients to another stage, where they either remained stable or continued down the natural progression of the disease or transitioned to an absorbent state of death, tooth loss, or blindness.

When the model allowed it, we also included outcomes of choosing different treatment pathways after detecting a lesion. In all cases, model validation was performed internally by varying key parameters to check how they may be associated with results and performing univariate and multivariate sensitivity analyses. All results were then compared with available research in their fields.

### Input Variables

**Figure 1.**
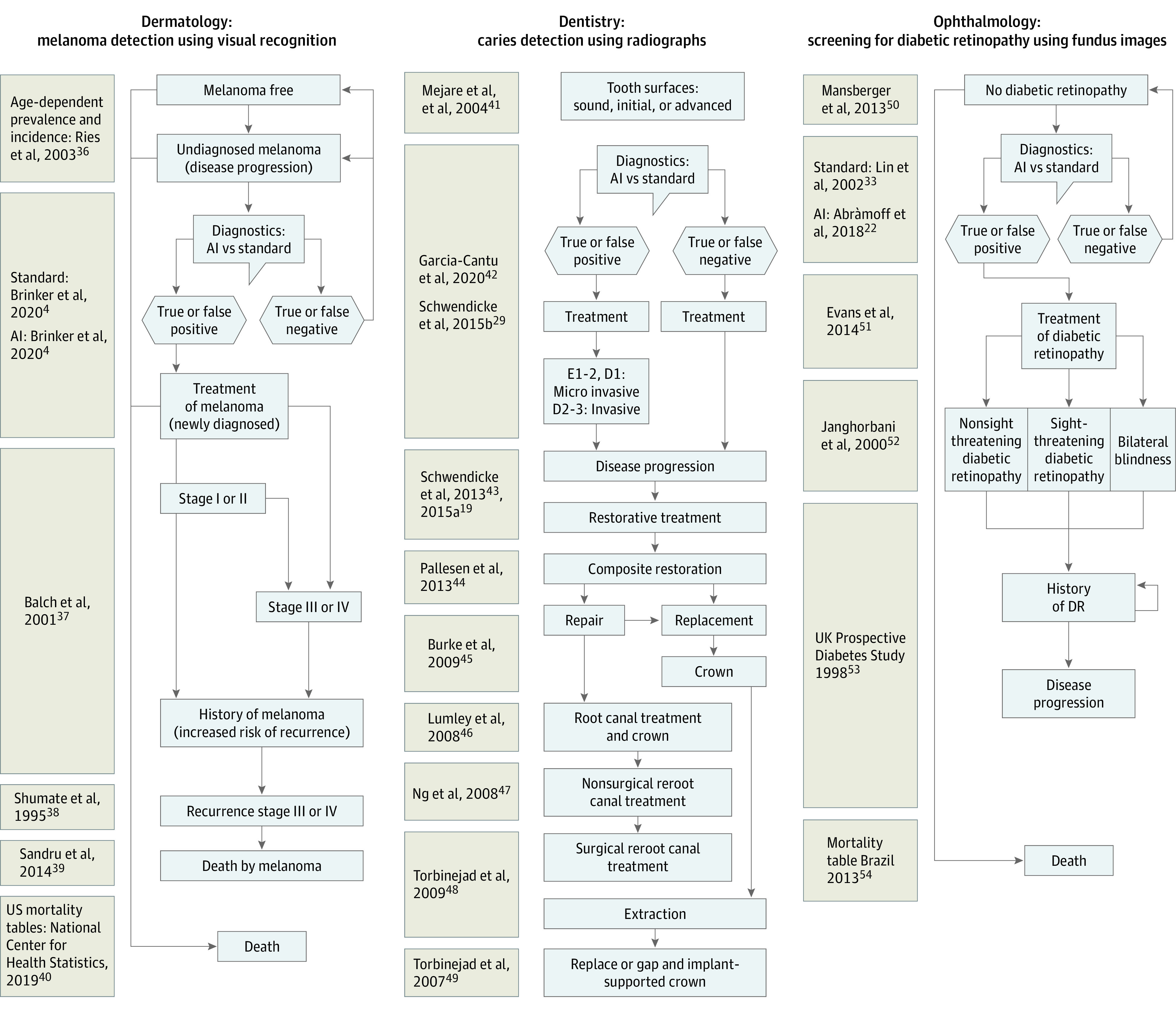
Visual Summary of the Different Models Included in the Study AI indicates artificial intelligence; DR, diabetic retinopathy.

Input variables were extracted from previous research used by the authors of the meta-analyses to construct their models. Diagnostic accuracies were also extracted from previous research. The references for the economic models and the diagnostic accuracy studies reporting on the different AI applications are summarized in Figure 1^4,19,22,29,33,36,37,38,39,40,41,42,43,44,45,46,47,48,49,50,51,52,53,54^ and the Table.^4,18,19,20,21,22^ Probabilities in prevalence rates, as well as sources, are described in eAppendices 1 through 3 in the Supplement.

### Health Outcomes, Costs, and Discounting

Health outcomes were expressed as quality-adjusted life years (QALYs) for the dermatology and ophthalmology use case and the mean time a tooth was retained (in years) for the dental use case. Cost calculations from a payer perspective were built on costs estimated out-of-pocket (OOP) from a patient perspective in the US case (ie, dermatological use case), a combination of prices extracted from the public catalog of services paid by statutory insurance and a catalog of private services in the German case (dental use case), and payer perspective in the Brazilian case (ophthalmological use case).

Costs for the application of AI were charged as a fee for service. For the dental use case, an €8 fee per application had been assumed in the original publication based on direct costs for research, development, operation, and overhead. We proceeded to charge the same amount in local currency in the other cases and then performed univariate sensitivity analysis.

Costs and tooth retention–years were discounted at 3% per annum in all 3 cases and variated in a univariate sensitivity analysis between 0% and 10%.^55^ Given our study’s perspective, opportunity costs were not accounted for.

### Statistical Analysis

We performed Monte Carlo microsimulations with 1000 independent individuals or teeth. Incremental cost-effectiveness ratios (ICERs) were used to express cost differences per QALY or mean year of tooth retention when comparing the 2 strategies. Results after performing 1000 Monte Carlo microsimulations with 1000 random samples in all 3 models can be found in the Table. To introduce parameter uncertainty, we randomly sampled transition probabilities from distributions reported in the original models and calculated 95% CIs or the range of parameters.^56^ In the case of caries progression, we used uniform distributions.

Using estimates for costs (in US dollars, euros, and Brazilian real) and years for dentistry and QALY for dermatology and ophthalmology, the net benefit of each strategy combination was calculated as a mean average of each cohort using the formula: individual net benefit = WTP × change in QALYs or tooth retention–years − change in cost, where WTP indicates the ceiling threshold of willingness to pay, ie, the additional costs a decision-maker is willing to bear for gaining an additional QALY or tooth retention–year.^32^ If WTP was greater than change in cost divided by the change in QALYs or tooth retention–years, an alternative intervention was considered more cost-effective than the comparator despite possibly being more costly.^56^ We used the net-benefit approach to calculate the probability of each intervention being acceptable regarding its cost-effectiveness for payers with different WTP ceiling thresholds. One-way sensitivity analyses were additionally performed to assess which strategy is associated with lowest cost or greatest increase in QALYs or tooth retention–years if key input parameters were changed to extreme values, thus exploring the impact of uncertainty and heterogeneity. Euros and reales were converted using 2020 Organisation for Economic Cooperation and Development purchasing power parities (PPP)^57^ at €0.746 and R $2.362 per US $1, respectively. Significant results were determined using 95% CIs with percentiles 2.5% and 97.5%. All analyses were undertaken using R2 Healthcare version 2.1 (TreeAge).

## Results

**Figure 2.**
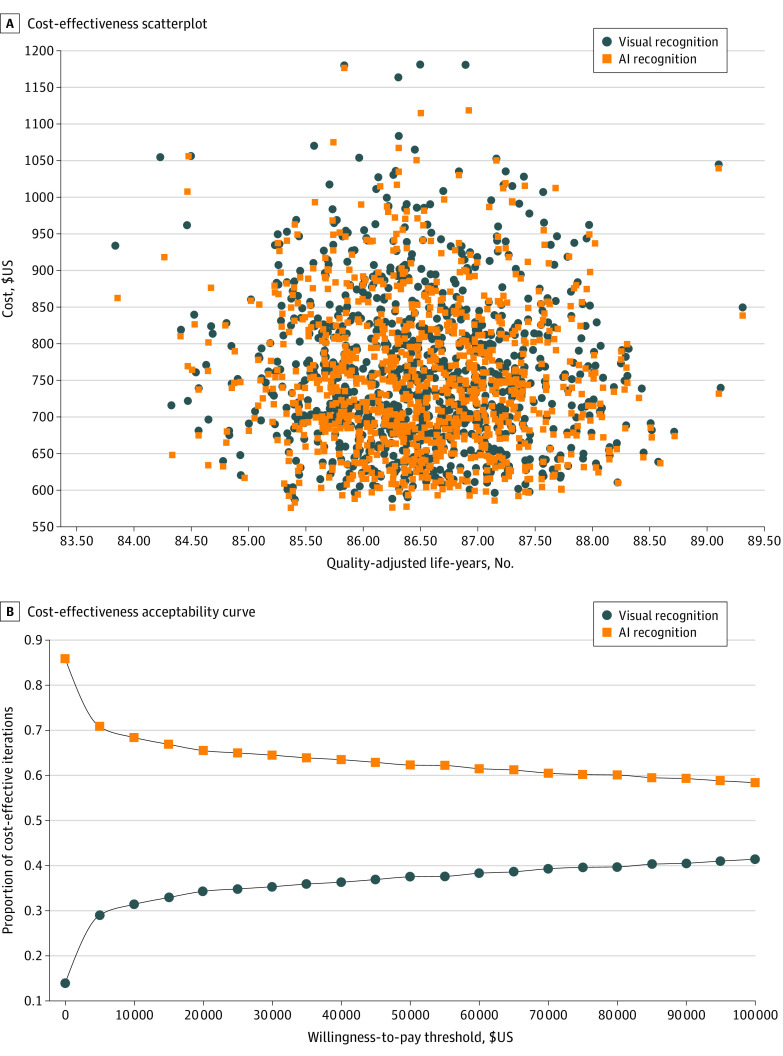
Cost-effectiveness of AI vs Standard of Care in Dermatology AI indicates artificial intelligence. In panel A, each dot and square represents an individual’s lifetime costs accrued (in US$) when receiving either standard of care (ie, visual recognition) or AI-assisted screening. In panel B, although AI is more likely to be cost-effective at a lower willingness to pay (WTP), these results show high sensitivity to WTP.

**Figure 3.**
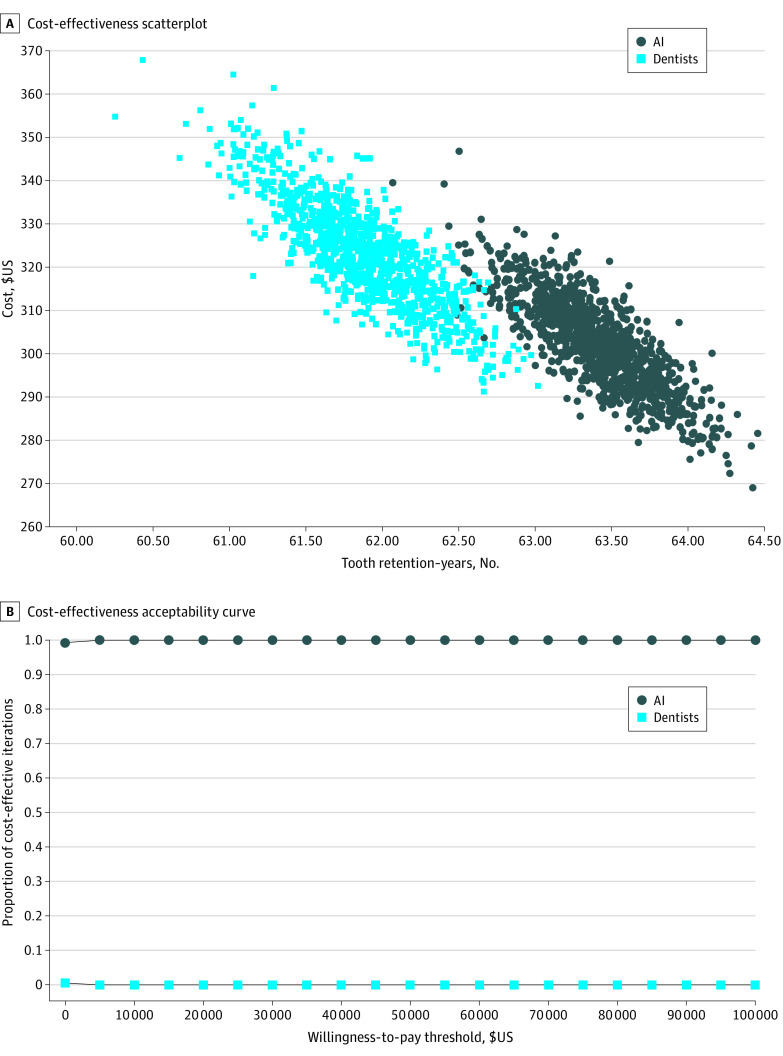
Cost-effectiveness of AI vs Standard of Care in Dentistry AI indicates artificial intelligence. In panel A, each dot and square represents a single tooth’s lifetime costs accrued (in euros) after receiving either standard of care diagnostics or AI-assisted screening. In panel B, AI is more likely to be cost-effective at a lower willingness to pay, yet these results do not seem to be altered when one assumes higher willingness to pay (WTP).

**Figure 4.**
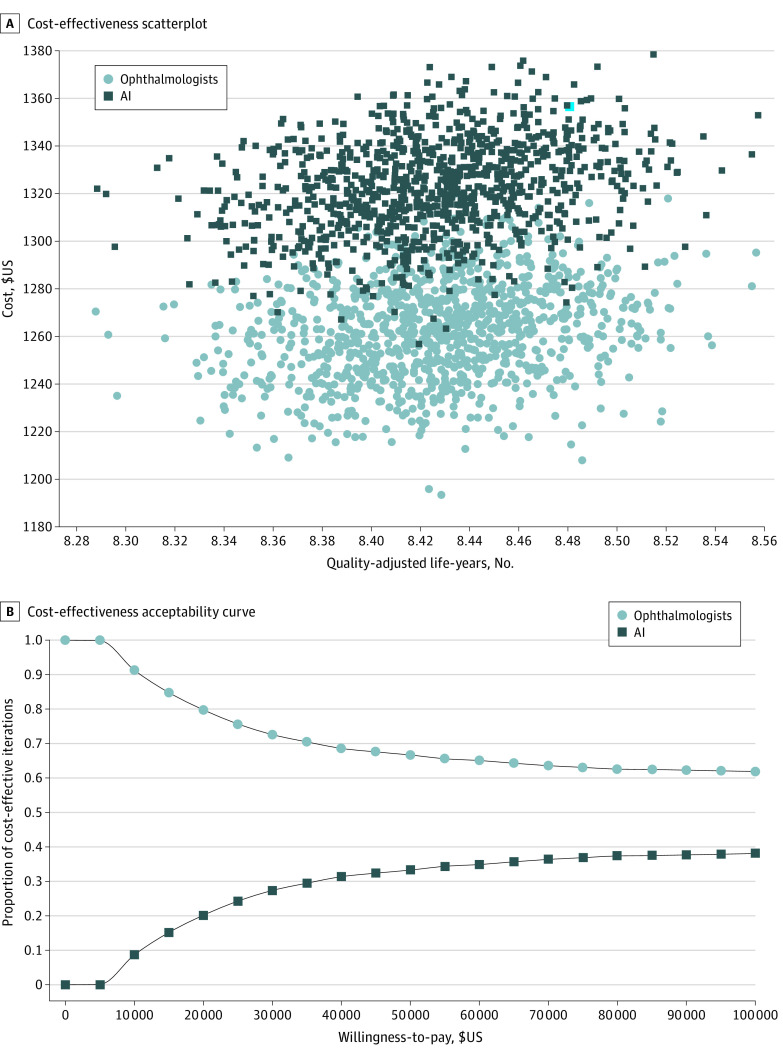
Cost-effectiveness of AI vs Standard of Care in Ophthalmology AI indicates artificial intelligence. In panel A, each dot and square represents a single individual’s lifetime costs accrued (Brazilian reales, R$) after receiving either standard of care diagnostics or AI-assisted screening. In panel B, AI was more likely to be less cost-effective at a lower willingness to pay based on study assumptions, although this certainty was sensitive to the willingness to pay (WTP) for additional quality-adjusted life years (QALYs).

In dermatology, the mean costs were $750 (95% CI, $608-$970) for AI and $759 (95% CI, $618-$980) for dermatologists without AI with similar health outcomes (AI, 86.6 QALYs; 95% CI, 84.9-88.0 QALYs; standard visual recognition, 86.6 QALYs; 95% CI, 84.9-88.0 QALYs). The ICER was −$27 580 per QALY (Figure 2A). The acceptability curve (Figure 2B) showed that AI was more likely to be more cost-effective at lower WTP; increasing WTP progressively increased the uncertainty (Figure 2B, Figure 3B, Figure 4B). Univariate sensitivity analysis on the discounting rates between 0% and 10% did not significantly affect results (eAppendix 4 in the Supplement). Univariate sensitivity analysis on the fee paid for the use of AI demonstrated that AI became the dominated strategy when the fee-for-service exceeded $16.

In dentistry, AI was associated with increased tooth retention (mean tooth retention, 62.4 years; 95% CI, 61.6-63.1 years) and less costly (€320; 95% CI, €299-€341) (US $429; 95% CI, $400-$458) than caries lesion detection without AI (mean tooth retention, 60.9 years; 95% CI, 61.5-63.1 years; cost, €342.24; €318-€368). The ICER was −€15.01 per year (US $20.12) (Figure 3A). The results were very sensitive to the treatment path modeled after diagnosis; when an invasive approach for detected lesions was considered, AI was associated with fewer years of tooth retention and higher cost. The acceptability curve shows that AI was more likely to be more cost-effective independent of the cost-effectiveness studied (Figure 3B). Univariate sensitivity analysis on discounting rates between 0% and 10% showed a dominance of AI over standard diagnostic methods when discounted rates remained below 6% (eAppendix 4 in the Supplement). Univariate sensitivity analysis on the fee paid for the use of AI demonstrated that AI became the dominated strategy when fee-for-service costs were above €16 (US $21.44).

In ophthalmology, the mean cost was R $1321 (95% CI, R $1283-R $1364) (US $559; 95% CI, US $543-$577) for AI and R $1260 (95% CI, R $1222-R $1303;) (US $533; 95% CI, $517-$551) for diagnosis without AI. Both strategies yielded a very similar mean (SD) utility of 8.4 (0.04) QALYs; however, AI increased costs by R $61 (US $25.82). The ICER was R −$91 760 (US −$38 848) (Figure 4A). The acceptability curve showed that standard of care was more likely to be more cost-effective, although higher WTP increased the uncertainty about the optimal strategy (Figure 4B).

Our results indicate that the incremental (per-person) cost per QALY would be R $39 705 (US $16 809); for reference, Brazilian GDP per capita PPP in 2020 was R $14 563 (US $6165). According to the thresholds recommended by the World Health Organization (WHO),^58^ the maximum cost paid per QALY gained could be up to 3 times the GDP per capita (in our example, R $43 689 [US $18 496]) to be considered cost-effective in these settings. The dominance of standard of care was not affected by a sensitivity analysis on the discounting rates (eAppendix 4 in the Supplement) nor by the price charged for the use of AI support (eAppendix 5 in the Supplement).

## Discussion

The cost-effectiveness of AI has been broadly studied and discussed for its potential to improve diagnosis,^14,59^ facilitate screening,^10,60^ and optimize laboratory tests and surgical appointments,^61,62^ among other use-cases.^63,64,65,66^ Our findings corroborate calls for solid economic evaluations of AI for health applications when AI is used to help determine care options for patients.^67^

To the best of our knowledge, this is the first study modeling several AI solutions against the standard of care. The main strength of this study was its design, which allowed comparisons of the same use case for the same technology used to detect different diseases. Our results suggest that the cost-effectiveness of AI vs standard of care should be evaluated specifically for each setting and use case, not only to consider the underlying costs generated by the AI application itself but also the treatments following diagnosis.

All AI solutions used as decision-support systems showed only moderate cost-effectiveness improvement. It can be assumed that if further improvements in AI are to be expected, its cost-effectiveness may improve too, as the accuracy of practitioner diagnosis without AI support is unlikely to increase. Moreover, regulation around AI, incentives for following AI recommendations, or differences in the efficiency and the diagnostic process when using AI or not should be explored further to come to a more realistic picture about the cost-effectiveness of AI in diagnostic support systems. Our results further indicate that AI may not necessarily have its biggest benefit in the hands of medical experts (where its advantages are limited) but could facilitate screening of patients in nonspecialist settings to allow targeted referral, as has been suggested in ophthalmology, for example.^59^ Evaluating these differences would require building new models and methods of evaluation, where higher magnitudes of effect may be expected.

The models included in our analysis were sensitive to the fee paid for the AI and only moderately affected by discounting rates. Our study suggests that small changes in the price can alter the dominance between strategies in this use case, making the economic impact of these digital tools sensitive to aspects of implementation, settings, payers perspectives, and use cases assumed. More research on different payment methods for AI will be necessary to allow robust comparisons and draw definitive conclusions on the health economic outcomes associated with AI technology as well as to determine the role AI could play in improving value-based care.

### Limitations

This study had several limitations. First, the limited information available on the research, operation and overhead costs, and payment mechanisms involved in incorporating AI did not allow for generating detailed comparisons. Aspects such as costs related to the hardware necessary for data acquisition were unknown and could potentially drastically alter our results. This uncertainty complicates establishing optimal pricing for AI services from a third-party payer perspective and is deserving of further scientific analysis. Regulations around subsequent treatment steps will also heavily affect overall cost-effectiveness and should be reflected in models. Regulators and decision-makers play an important role in making sure that developed AI solutions remain safe for patients and help to improve outcomes, while also sufficiently incentivizing further development so that digital health can accomplish some of the expectations it has generated.^57,58^ Analyzing real-world evidence after improvements in diagnostic technology enter the market seems a judicious approach to prioritize patient and clinical cost-effectiveness, and can clarify how improvements in diagnostic accuracy can impact the cost-effectiveness of AI. Future studies could consider the expected value of information analysis to assess the relevance of uncertainty of a range of parameters, including diagnostic accuracy, and steer research and development accordingly.

Second, it is important to recognize that differences in outcomes across our models could be due to inconsistencies in the use of AI between different income settings. Epidemiological factors and lower fee-for-services paid in low- and middle-income countries should be studied to avoid that AI does not worsen existing health inequalities. This fact calls for a better understanding of how epidemiological differences such as incidence and morbidity of a certain disease can factor into decisions to reimburse AI services. Because of that, future research could focus on developing analytical frameworks to facilitate comparisons of AI from different perspectives, in different settings, and for different outcomes. This could allow for more targeted development of AI solutions for use cases where they are most impactful and cost-effective.

Third, we assumed physicians would act according to the AI-detection results, ie, in perfect congruence. However, this is not a given—physicians may disagree with AI diagnoses and make decisions that alter the resulting diagnostic accuracy (both to the benefit or detriment of the resulting composite accuracy). The same applies for the resulting therapies. We therefore invite readers to consider our results as a base case scenario, as in practice deviations from our findings are likely. New studies assessing how physicians interact with software would be fundamental for understanding how AI could best synergize with medical practitioners.

## Conclusions

In this economic evaluation, AI used as a decision-support system came with limited and use case–specific cost-effectiveness advantages, which were sensitive not only to the costs assigned to AI but also the subsequent therapy paths assumed after the diagnosis. AI developers need to work jointly with regulators and the medical community to make sure that new AI solutions are deployed where they best improve outcomes. Developing appropriate payment mechanisms seems fundamental to incentivize new cost-effective therapies with this technology.

## Notes

Supplement. **eTable 1.** Input Parameters—Dermatology **eTable 2.** Input Parameters—Dentistry **eTable 3.** Input Parameters—Ophthalmology **eTable 4.** Sensitivity Analysis—Discounting Rate **eTable 5.** Sensitivity Analysis—Cost of AI
eReferences.
