# Supplementary material for: Cost-effectiveness of Artificial Intelligence as a Decision-Support System Applied to the Detection and Grading of Melanoma, Dental Caries, and Diabetic Retinopathy
Source: JAMA Netw Open. 2022 Mar 15;5(3):e220269. doi: 10.1001/jamanetworkopen.2022.0269 (PMC8924723; doi:10.1001/jamanetworkopen.2022.0269)
Supplement: Supplement. — eTable 1. Input Parameters—Dermatology eTable 2. Input Parameters—Dentistry eTable 3. Input Parameters—Ophthalmology eTable 4. Sensitivity Analysis—Discounting Rate eTable 5. Sensitivity Analysis—Cost of AI eReferences. [file jamanetwopen-e220269-s001.pdf]

Supplemental Online Content

Gomez Rossi J, Rojas-Perilla N, Krois J, Schwendicke F. Cost-effectiveness of artificial intelligence as a decision-support system applied to the detection and grading of melanoma, dental caries, and diabetic retinopathy. *JAMA Netw Open.* 2022;5(3):e220269. doi:10.1001/jamanetworkopen.2022.0269

eTable 1. Input Parameters—Dermatology

eTable 2. Input Parameters—Dentistry

eTable 3. Input Parameters—Ophthalmology

eTable 4. Sensitivity Analysis—Discounting Rate

eTable 5. Sensitivity Analysis—Cost of AI

eReferences.

This supplemental material has been provided by the authors to give readers additional information about their work.

eTable 1. Input parameters—Dermatology

**Prevalence, accuracy, lesion development and progression**				
**Estimate**	**Source (reference)**	**Values**		
Sensitivity<br>Standard	Brinker et al. (4)	0.894		
Specificity<br>Standard	Brinker et al. (4)	0.644		
Sensitivity<br>AI diagnostic	Brinker et al. (4)	0.894		
Specificity<br>AI diagnostic	Brinker et al. (4)	0.682		
Probability of being tested at any cycle	Losina et al. (25)	0.05		
Probability of lesion development (Incidence)	Balch et al. 2001 (41)	0.00196		
If undiagnosed, progression rate to next stage	Tsao et al. (42)	0.1		
If disease has been diagnosed and treated, the risk of disease progression to next stage	Shumate et al. 1995(43)	(Table obtained from 19) -		
**Health state**	**Source (reference)**	**Transition probability per cycle**	**Transition to**	**Health state utility**
Stage I	Balch et al. 2001 (41)	0.90 (0.81 – 0.99)	Disease progression<br>Remain undiagnosed<br>Screening (Standard or AI)<br>Death	0.937
Stage II	Balch et al. 2001 (41)	0.72 (0.65 – 0.79)	Disease progression<br>Remain undiagnosed<br>Screening (Standard or AI)<br>Death	0.937
Stage III	Balch et al. 2001 (41)	0.5 (0.45 – 0.55)	Disease progression<br>Remain undiagnosed<br>Screening (Standard or AI)<br>Death	0.52
Stage IV	Balch et al. 2001 (41)	0.13 (0.11 – 0.15)	Disease progression<br>Remain undiagnosed<br>Screening (Standard or AI)<br>Death to Melanoma<br>Death	0.52
History of melanoma- Stage I, II, III, IV	Svedman et al. 2016 (19)	Table is taken from Svedman et al. (19)	Disease progression<br>Remain in the same stage<br>(Death to Melanoma)<br>Death	

© 2022 Rossi JG et al. *JAMA Network Open.*

Death	2007 Mortality table (45)	Table taken from (21)	Death	0

Sensitivities and specificities of disease detection with and without AI assistance were derived from the economic analysis and compared with the existing meta-analysis. If possible, we calculated mean values and 95% confidence intervals or ranges to estimate normal distributions (in parentheses) for random sampling during microsimulation.

© 2022 Rossi JG et al. *JAMA Network Open.*

eTable 2. Input parameters—Dentistry

Prevalence, accuracy, lesion development and progression				
**Estimate**	**Source (reference)**	**Initial lesions (up to inner third of enamel) (E2)**	**Initial lesions (up outer third of dentin) (D1)**	**Advanced lesions (middle third of dentin) (D2)**
**Prevalence**				
Low risk	Schwendicke et al. (23)	0.14	0.025	0.005
High risk	Schwendicke et al. (23)	2.14 × 0.14	1.66 × 0.025	1.66 × 0.005
**Sensitivity and specificity**				
Sensitivity<br>Visual-tactile	Schwendicke et al. (23)	0.00	0.00	0.311 (0.270-0.353)
Specificity<br>Visual-tactile	Schwendicke et al. (23)	1.00	1.00	0.922 (0.892-0.945)
Sensitivity<br>Radiography w/o AI (control)*	Schwendicke et al. (38)	0.24 (0.21-0.26)	0.36 (0.24-0.49)	0.64 (0.59-0.70)
Specificity<br>Radiography w/o AI (control)*	Schwendicke et al. (38)	0.97 (0.95-0.98)	0.94 (0.89-0.97)	0.98 (0.97-0.98)
Sensitivity<br>Radiography w AI (test)	Garcia Cantu et al. (39)	0.68	0.68	0.58
Specificity<br>Radiography w AI (test)	Garcia Cantu et al. (39)	0.86	0.86	0.96
Probability of being tested	KZBV- Statutory Guideline (46)	2 per year	2 per year	2 per year
Probability of lesion development	Schwendicke et al. (23)	$p=1.26 \times 0.57252 \times 2.7^{-0.1472 \times 2\alpha}$<br>distribution: 1.24-1.29	$p=1.26 \times 0.0426 \times 2.7^{-0.0521 \times 2\alpha}$<br>distribution: 1.24-1.29	$p=1.26 \times 0.57 \times 0.0426 \times 2.7^{-0.0521 \times 2\alpha}$<br>distribution: 1.24-1.29
Probability of lesion progression				
Progression to		D1 lesion	D2 lesion	D3 lesion
If untreated	Schwendicke et al. (23)	p=2.63 (high risk) / 2.13 (low risk) × $3.0984\times(2\alpha)^{-1.343}$<br>(distribution: p × 0.87 – p × 1.13)	p=2.63 (high risk) / 2.13 (low risk) × $161.52\times(2\alpha)^{-2.078}$<br>(distribution: p × 0.87 – p × 1.13)	$p=1.32 \times 161.52 \times (2\alpha)^{-2.078}$<br>(distribution: p × 0.87 – p × 1.13)
If infiltrated	Schwendicke et (23)	$p=0.4289\times(2\alpha)^{-1.391}$<br>(distribution: p × 0.23 – p × 5.15)	$p=68.869\times(2\alpha)^{-2.078}$<br>(distribution: p × 0.23 – p × 4.17)	-
**Transition probabilities**				
**Health state**	**Source (reference)**	**Transition probability per cycle**	**Transition to**	**Allocation probability**
Composite[11]	(Pallesen et al.)	0.016	Composite<br>Crown<br>Repair<br>Rootcanal treatment	0.45<br>0.10<br>0.10<br>0.25

© 2022 Rossi JG et al. *JAMA Network Open.*

			Extraction	0.10
Direct capping[2]	Schwendicke et al. (47)	0.111	Rootcanal treatment<br>Extraction	0.95<br>0.05
Crown on vital tooth[3]	Burke and Lucarotti (48)	0.036	Rootcanal treatment<br>Recementation<br>Repair<br>Re- crown<br>Extraction	0.25<br>0.15<br>0.10<br>0.40<br>0.10
Root canal treatment	Lumley et al. (49)	0.021	Non-surgical re-treatment<br>Surgical re-treatment<br>Extraction	0.20<br>0.30<br>0.50
Crown on non-vital tooth[3]	Burke and Lucarotti (48)	0.029	Recementation<br>Repair<br>Re- crown[3]<br>Extraction	0.20<br>0.10<br>0.60<br>0.10
Non-surgical root-canal treatment	Ng et al. (50)	0.085(Ng et al. 2008)	Surgical re-treatment<br>Extraction	0.25<br>0.75
Surgical root-canal treatment	Torabinejad et al. (51)	0.061	Extraction	1.00
Implant and implant-supported crown	Torabinejad et al. (52)	0.010	Recementation/refixing<br>Re-crown<br>Re-implant	0.60<br>0.20<br>0.20

[1] Data from 15-19-year-olds. The risk of pulpal exposure during re-composite assumed to be 10%. Crowning assumed if re-restored before.

[2] 95% of exposed pulps were treated using direct capping, 5% were assumed to receive immediate root canal treatment.

[3] For non-vital crowned teeth, the risk of endodontic complications was calculated separately (Ferrari et al. 2012).

Analyses were performed for populations with low and high caries prevalence and risks, respectively. Sensitivities and specificities of caries detection with and without AI assistance were derived from our primary study and a meta-analysis as described. The probabilities of lesion development and progression if untreated or infiltrated were calculated according to the patient's age (α) using hazard functions. If possible, we calculated mean values and 95% confidence intervals or ranges to estimate distributions (in parentheses) for random sampling during microsimulation.

© 2022 Rossi JG et al. *JAMA Network Open.*

eTable 3. Input parameters—Ophthalmology

Prevalence, accuracy, disease development and progression			
**Estimate**	**Source (reference)**	**Values**	
Type II diabetes, prevalence	Mansberg et al. (53)	0.14 (0.09-0.25)	
**Sensitivity and specificity**			
Sensitivity Standard	Lin et al. (27)	0.829 (0.805-0.85)	
Specificity Standard		0.92 (0.88-0.96)	
Sensitivity AI diagnostic	Abramoff et al. (40)	0.87 (0.81-0.91)	
Specificity AI diagnostic		0.91 (0.83-0.93)	
Probability of being tested	An et al. (54)	0.50 (0.15-0.90)	
**Utility values**			
No diabetic retinopathy (No-DR)	Ben et al. (55)	*0.748 (0.698-0.798)	
Non-sight threatening diabetic retinopathy (Non-STDR)		0.752 (0.679-0.825)	
Sight threatening diabetic retinopathy (STDR)		0.628 (0.521-0.726)	
Bilateral blindness (BB)		0.355 (0.105-0.606)	
**Treatment effects**			
Risk reduction of worsening using laser treatment	Evans et al. (56)	0.49 (0.37-0.64)	
**Transition probabilities**			
**Health state**	**Source (reference)**	**Transition to**	**Transition probability per cycle**
No-DR to	Janghorbani et al. / UKDPS(57,58)	Non-STDR<br>STDR<br>BB	0.0541(0.0516–0.0555)<br>0.0541(0.0516–0.0555)<br>0.0016 (0.0014–0.0020)
Non-STDR		STDR<br>BB	0.0541(0.0516–0.0555)<br>0.0040(0.0034–0.0050)
STDR		BB	0.0080 (0.0068–0.0090)
Death	Brazilian ministry of health(59)	Death	Table 2013
**Annual costs/ state**	**Source (Reference)**	**Cost**	
From NoDR to other states	Ben et al. (55), Brazilian ministry of health (60)	R$16 (R$14–R$19)	
From NoDR/ Non-STDR to STDR		R$122 (R$104–R$140)	
From NoDR/ Non-STDR to BB		R$15 (R$12–R$17)	

© 2022 Rossi JG et al. *JAMA Network Open.*

From STDR to BB		R$79 (R$67–R$91)
Cost Non-STDR		R$235 (R$199–R$270)
Cost STDR		R$15 (R$12–R$17)
Cost BB		R$37 (R$31–R$43)
Cost AI screening	(16)	R$8 (R$0–R$40)

Sensitivities and specificities of disease detection with and without AI assistance were derived from the economic analysis and compared with the existing meta-analysis. If possible, we calculated mean values and 95% confidence intervals or ranges for random sampling during microsimulation.

*The population modelled consists of already diabetic patients, reason why utility even without DR is never 1. Transition probability to other stages can be sudden, however, patients are assumed to already accrue costs for their standard treatment.

© 2022 Rossi JG et al. *JAMA Network Open.*

eTable 4. Sensitivity analysis—Discounting rate

Dermatology

Discount Rate	Strategy	Strategy Index	Cost	Inc Cost	Eff	Increase Eff	ICER	NMB	C/E	Dominance
0	AI recognition	1	731.93	0	87.25	0	0	2.62E+07	8.38	
0	Visual recognition	0	738.50	6.57	87.23	-0.015	-412.64	2.62E+07	8.46	(Dominated)
0.01	Visual recognition	0	678.21	0	69.63	0	0	2.09E+07	9.73	
0.01	AI recognition	1	679.75	1.53	69.64	0.010	141.89	2.09E+07	9.76	
0.02	Visual recognition	0	634.46	0	56.78	0	0	1.70E+07	11.17	
0.02	AI recognition	1	641.94	7.47	56.79	0.007	1010.29	1.70E+07	11.30	
0.03	Visual recognition	0	602.14	0	47.25	0	0	1.42E+07	12.74	
0.03	AI recognition	1	614.04	11.90	47.25	0.0051	2333.74	1.42E+07	12.99	
0.04	Visual recognition	0	577.85	0	40.04	0	0	1.20E+07	14.42	
0.04	AI recognition	1	593.11	15.25	40.04	0.0035	4306.27	1.20E+07	14.80	
0.05	Visual recognition	0	559.28	0	34.50	0	0	1.04E+07	16.20	
0.05	AI recognition	1	577.12	17.83	34.50	0.0024	7189.68	1.04E+07	16.72	
0.06	Visual recognition	0	544.87	0	30.17	0	0	9051062	18.059	
0.06	AI recognition	1	564.73	19.85	30.17	0.0017	11329.3	9051568	18.71	
0.07	Visual recognition	0	533.52	0	26.72	0	0	8018135	19.96	
0.07	AI recognition	1	554.98	21.46	26.73	0.00125	17168.06	8018488	20.76	
0.08	Visual recognition	0	524.46	0	23.95	0	0	7184593	21.89	
0.08	AI recognition	1	547.21	22.75	23.95	9.01E-04	25253.64	7184840	22.84	
0.09	Visual recognition	0	517.13	0	21.67	0	0	6502180	23.85	
0.09	AI recognition	1	540.94	23.80	21.67	6.57E-04	36230.48	6502353	24.95	
0.1	Visual recognition	0	511.14	0	19.78	0	0	5935946	25.83	
0.1	AI recognition	1	535.82	24.67	19.78	4.86E-04	50805.69	5936067	27.07	

© 2022 Rossi JG et al. *JAMA Network Open.*

Dentistry

Discount Rate	Strategy	Strategy Index	Cost	Incr Cost	Eff	Incr Eff	ICER	NMB	C/E	Dominance
0.01	AI	0	552.72	0	62.35	0	0	-552.73	8.86	
0.01	Dentists	1	620.61	67.88	60.91	-1.44	-46.95	-620.61	10.18	(Dominated)
0.019	AI	0	421.33	0	62.35	0	0	-421.33	6.75	
0.019	Dentists	1	462.61	41.28	60.91	-1.44	-28.55	-462.61	7.59	(Dominated)
0.028	AI	0	335.92	0	62.35	0	0	-335.92	5.38	
0.028	Dentists	1	360.63	24.70	60.91	-1.44	-17.08	-360.63	5.92	(Dominated)
0.037	AI	0	277.52	0	62.35	0	0	-277.52	4.45	
0.037	Dentists	1	291.28	13.75	60.91	-1.44	-9.51	-291.28	4.78	(Dominated)
0.046	AI	0	235.91	0	62.35	0	0	-235.91	3.78	
0.046	Dentists	1	242.09	6.18	60.91	-1.44	-4.28	-242.1	3.97	(Dominated)
0.055	AI	0	205.20	0	62.35	0	0	-205.20	3.29	
0.055	Dentists	1	205.98	0.77	60.91	-1.44	-0.53	-205.98	3.38	(Dominated)
0.064	Dentists	1	178.65	0	60.91	0	0	-178.65	2.93	
0.064	AI	0	181.86	3.21	62.35	1.44	2.22	-181.87	2.91	
0.073	Dentists	1	157.44	0	60.91	0	0	-157.44	2.58	
0.073	AI	0	163.65	6.21	62.35	1.44	4.29	-163.66	2.62	
0.082	Dentists	1	140.59	0	60.91	0	0	-140.6	2.30	
0.082	AI	0	149.11	8.51	62.35	1.44	5.89	-149.11	2.39	
0.091	Dentists	1	126.95	0	60.91	0	0	-126.95	2.08	
0.091	AI	0	137.26	10.31	62.35	1.44	7.13	-137.26	2.20	
0.1	Dentists	1	115.70	0	60.91	0	0	-115.70	1.89	
0.1	AI	0	127.43	11.73	62.35	1.44	8.11	-127.43	2.04	

© 2022 Rossi JG et al. *JAMA Network Open.*

Ophthalmology

Discount Rate	Strategy	Strategy Index	Cost	Incr Cost	Eff	Incr Eff	ICER	NMB	C/E	Dominance
0	Ophthalmologist	0	2871.83	0	15.08	0	0	-2871.83	190.43	
0	Screening AI	1	2956.04	84.216	15.07	-0.00251	-33591.6	-2956.05	196.05	(Dominated)
0.01	Ophthalmologist	0	2380.24	0	13.12	0	0	-2380.25	181.34	
0.01	Screening AI	1	2458.09	77.844	13.12	-0.00202	-38536.6	-2458.09	187.30	(Dominated)
0.02	Ophthalmologist	0	1996.99	0	11.56	0	0	-1997	172.66	
0.02	Screening AI	1	2069.50	72.504	11.56	-0.00164	-44162.4	-2069.5	178.95	(Dominated)
0.03	Ophthalmologist	0	1694.97	0	10.30	0	0	-1694.98	164.46	
0.03	Screening AI	1	1762.97	67.992	10.30	-0.00135	-50541.5	-1762.97	171.08	(Dominated)
0.04	Ophthalmologist	0	1454.45	0	9.27	0	0	-1454.45	156.78	
0.04	Screening AI	1	1518.59	64.148	9.27	-0.00111	-57753.7	-1518.6	163.71	(Dominated)
0.05	Ophthalmologist	0	1260.90	0	8.42	0	0	-1260.91	149.64	
0.05	Screening AI	1	1321.76	60.851	8.42	-9.24E-04	-65888.4	-1321.76	156.88	(Dominated)
0.06	Ophthalmologist	0	1103.60	0	7.71	0	0	-1103.61	143.03	
0.06	Screening AI	1	1161.60	58.001	7.71	-7.73E-04	-75045.3	-1161.61	150.56	(Dominated)
0.07	Ophthalmologist	0	974.51	0	7.11	0	0	-974.51	136.94	
0.07	Screening AI	1	1030.03	55.524	7.11	-6.51E-04	-85337.5	-1030.04	144.75	(Dominated)
0.08	Ophthalmologist	0	867.58	0	6.60	0	0	-867.58	131.33	
0.08	Screening AI	1	920.93	53.357	6.60	-5.51E-04	-96893.9	-920.93	139.42	(Dominated)
0.09	Ophthalmologist	0	778.21	0	6.16	0	0	-778.21	126.19	
0.09	Screening AI	1	829.66	51.450	6.16	-4.68E-04	-109862	-829.66	134.54	(Dominated)
0.1	Ophthalmologist	0	702.89	0	5.78	0	0	-702.89	121.47	
0.1	Screening AI	1	752.65	49.764	5.78	-4.00E-04	-124412	-752.65	130.08	(Dominated)

© 2022 Rossi JG et al. *JAMA Network Open.*

eTable 5. Sensitivity analysis—Cost of AI

Dermatology

AI costs (US$)	Base case	Strategy	Strategy Index	Cost	Incr Cost	Eff	Incr Eff	ICER	NMB	C/E	Dominance
0		AI recognition	1	727.99	0	86.62	0	0	2.60E+07	8.40	
0		Visual recognition	0	762.32	34.33	86.62	0.0015	22350.23	2.60E+07	8.80	
4		AI recognition	1	736.90	0	86.62	0	0	2.60E+07	8.50	
4		Visual recognition	0	762.32	25.42	86.62	0.0015	16550.96	2.60E+07	8.80	
8	Base case	AI recognition	1	745.81	0	86.62	0	0	2.60E+07	8.61	
8	Base case	Visual recognition	0	762.32	16.51	86.62	0.0015	10751.69	2.60E+07	8.80	
12		AI recognition	1	754.72	0	86.62	0	0	2.60E+07	8.71	
12		Visual recognition	0	762.32	7.60	86.62	0.0015	4952.42	2.60E+07	8.80	
16		Visual recognition	0	762.32	0	86.62	0	0	2.60E+07	8.80	
16		AI recognition	1	763.63	1.30	86.62	-0.0015	-846.85	2.60E+07	8.81	(Dominated)
20		Visual recognition	0	762.32	0	86.62	0	0	2.60E+07	8.80	
20		AI recognition	1	772.54	10.21	86.62	-0.0015	-6646.12	2.60E+07	8.91	(Dominated)
24		Visual recognition	0	762.32	0	86.62	0	0	2.60E+07	8.80	
24		AI recognition	1	781.45	19.12	86.62	-0.0015	-12445.4	2.60E+07	9.02	(Dominated)
28		Visual recognition	0	762.32	0	86.62	0	0	2.60E+07	8.80	
28		AI recognition	1	790.36	28.03	86.62	-0.0015	-18244.7	2.60E+07	9.12	(Dominated)
32		Visual recognition	0	762.32	0	86.62	0	0	2.60E+07	8.80	
32		AI recognition	1	799.27	36.94	86.62	-0.0015	-24043.9	2.60E+07	9.22	(Dominated)
36		Visual recognition	0	762.32	0	86.62	0	0	2.60E+07	8.80	
36		AI recognition	1	808.18	45.85	86.62	-0.0015	-29843.2	2.60E+07	9.33	(Dominated)
40		Visual recognition	0	762.32	0	86.62	0	0	2.60E+07	8.80	

© 2022 Rossi JG et al. *JAMA Network Open.*

40		AI recognition	1	817.09	54.76	86.62	-0.0015	-35642.5	2.60E+07	9.43	(Dominated)

Dentistry

AI costs (Euro)	AI costs PPP International $ 2020 OECD	Base case	Strategy	Strategy Index	Cost	Incr Cost	Eff	Incr Eff	ICER	NMB	C/E	Dominance
0	0		AI	0	299.93	0	63.40	0	0	-299.93	4.73	
0	0		Dentists	1	324.04	24.10	61.85	-1.55	-15.54	-324.04	5.23	(Dominated)
4	3		Dentists	1	324.04	0	61.85	0	0	-324.04	5.23	
4	3		AI	0	336.89	12.85	63.40	1.55	8.29	-336.89	5.31	
**8**	**6**	**Base case**	Dentists	1	324.04	0	61.85	0	0	-324.04	5.23	
**8**	**6**	**Base case**	AI	0	373.86	49.82	63.40	1.55	32.13	-373.86	5.89	
12	9		Dentists	1	324.04	0	61.85	0	0	-324.04	5.23	
12	9		AI	0	410.83	86.79	63.40	1.55	55.97	-410.83	6.47	
16	12		Dentists	1	324.04	0	61.85	0	0	-324.04	5.23	
16	12		AI	0	447.8	123.75	63.40	1.55	79.81	-447.8	7.06	
20	15		Dentists	1	324.04	0	61.85	0	0	-324.04	5.23	
20	15		AI	0	484.76	160.72	63.40	1.55	103.65	-484.76	7.64	
24	18		Dentists	1	324.04	0	61.85	0	0	-324.04	5.23	
24	18		AI	0	521.73	197.69	63.40	1.55	127.49	-521.73	8.22	
28	21		Dentists	1	324.04	0	61.85	0	0	-324.04	5.23	
28	21		AI	0	558.70	234.65	63.40	1.55	151.33	-558.70	8.81	
32	24		Dentists	1	324.04	0	61.85	0	0	-324.04	5.23	
32	24		AI	0	595.66	271.62	63.40	1.55	175.17	-595.66	9.39	
36	27		Dentists	1	324.04	0	61.85	0	0	-324.04	5.23	
36	27		AI	0	632.63	308.59	63.40	1.55	199.01	-632.63	9.97	
40	30		Dentists	1	324.04	0	61.85	0	0	-324.04	5.23	
40	30		AI	0	669.60	345.56	63.40	1.55	222.85	-669.60	10.55	

© 2022 Rossi JG et al. *JAMA Network Open.*

Opthalmology

AI costs (Reales)	AI costs PPP International $ 2020 OECD	Base case	Strategy	Strategy Index	Cost	Incr Cost	Eff	Incr Eff	ICER	NMB	C/E	Dominance
0	0		Ophthalmologist	0	1260.90	0	8.42	0	0	-1260.91	149.64	
0	0		Screening AI	1	1294.89	33.98	8.42	-9.24E-04	-36800.2	-1294.9	153.69	(Dominated)
4	1,73		Ophthalmologist	0	1260.90	0	8.42	0	0	-1260.91	149.64	
4	1,73		Screening AI	1	1308.32	47.41	8.42	-9.24E-04	-51344.3	-1308.33	155.28	(Dominated)
**8**	**3,46**	**Base case**	Ophthalmologist	0	1260.90	0	8.42	0	0	-1260.91	149.64	
**8**	**3,46**	**Base case**	Screening AI	1	1321.76	60.85	8.42	-9.24E-04	-65888.4	-1321.76	156.88	(Dominated)
12	5,19		Ophthalmologist	0	1260.90	0	8.42	0	0	-1260.91	149.64	
12	5,19		Screening AI	1	1335.19	74.28	8.42	-9.24E-04	-80432.4	-1335.19	158.47	(Dominated)
16	6,92		Ophthalmologist	0	1260.90	0	8.42	0	0	-1260.91	149.64	
16	6,92		Screening AI	1	1348.62	87.71	8.42	-9.24E-04	-94976.5	-1348.62	160.06	(Dominated)
20	8,65		Ophthalmologist	0	1260.90	0	8.42	0	0	-1260.91	149.64	
20	8,65		Screening AI	1	1362.05	101.14	8.42	-9.24E-04	-109521	-1362.06	161.66	(Dominated)
24	10,39		Ophthalmologist	0	1260.90	0	8.42	0	0	-1260.91	149.64	
24	10,39		Screening AI	1	1375.48	114.57	8.42	-9.24E-04	-124065	-1375.49	163.25	(Dominated)
28	12,12		Ophthalmologist	0	1260.90	0	8.42	0	0	-1260.91	149.64	
28	12,12		Screening AI	1	1388.92	128.01	8.42	-9.24E-04	-138609	-1388.92	164.85	(Dominated)
32	13,85		Ophthalmologist	0	1260.90	0	8.42	0	0	-1260.91	149.64	
32	13,85		Screening AI	1	1402.35	141.44	8.42	-9.24E-04	-153153	-1402.35	166.44	(Dominated)
36	15,58		Ophthalmologist	0	1260.90	0	8.42	0	0	-1260.91	149.64	
36	15,58		Screening AI	1	1415.78	154.87	8.42	-9.24E-04	-167697	-1415.79	168.03	(Dominated)
40	17,31		Ophthalmologist	0	1260.90	0	8.42	0	0	-1260.91	149.64	
40	17,31		Screening AI	1	1429.21	168.30	8.42	-9.24E-04	-182241	-1429.22	169.63	(Dominated)

© 2022 Rossi JG et al. *JAMA Network Open.*

eReferences.

An J, Niu F, Turpcu A, Rajput Y, Cheetham TC. Adherence to the American Diabetes Association retinal screening guidelines for population with diabetes in the United States. *Ophthalmic Epidemiol*. 2018;25(3):257-265. Medline:29333897 doi:10.1080/09286586.2018.1424344

Ben ÂJ, Souza CF, de, et al. Health-related quality of life associated with diabetic retinopathy in patients at a public primary care service in southern Brazil. *Arch Endocrinol Metab*. 2021;64(5):575-583. Medline:34033298 doi:10.20945/2359-3997000000223

National Association of German Dentists website. Bonusheft. Accessed March 23, 2021. https://www.kzbv.de/bonusheft.39.de.html

National Institute of Statistics, Brazil. Medicamentos e OPM do SUS [Mortality table]. Updated November 25, 2021. Accessed April 28, 2021. http://sigtap.datasus.gov.br/tabela-unificada/app/sec/inicio.jsp

Svedman FC, Pillas D, Taylor A, Kaur M, Linder R, Hansson J. Stage-specific survival and recurrence in patients with cutaneous malignant melanoma in Europe—a systematic review of the literature. *Clin Epidemiol*. 2016;8:109-122. Medline:27307765 doi:10.2147/CLEP.S99021

Tsao H, Rogers GS, Sober AJ. An estimate of the annual direct cost of treating cutaneous melanoma. *J Am Acad Dermatol*. 1998;38(5 Pt 1):669-680. Medline:9591809 doi:10.1016/S0190-9622(98)70195-1

US Food and Drug Administration. Artificial Intelligence and Machine Learning in Software as a Medical Device. FDA website. Updated September 22, 2021. Accessed February 25, 2021. https://www.fda.gov/medical-devices/software-medical-device-samd/artificial-intelligence-and-machine-learning-software-medical-device

US Food and Drug Administration. Digital Health Center of Excellence. FDA website. Updated January 27, 2022. Accessed February 25, 2021. https://www.fda.gov/medical-devices/digital-health-center-excellence

© 2022 Rossi JG et al. *JAMA Network Open.*
